# Hörklassen bei Patienten mit Vestibularisschwannom bei Verwendung deutschsprachiger Testverfahren

**DOI:** 10.1007/s00106-020-00948-4

**Published:** 2020-09-25

**Authors:** T. Rahne, S. K. Plontke, D. Vordermark, C. Strauss, C. Scheller

**Affiliations:** 1 Universitätsklinik und Poliklinik für Hals‑, Nasen‑, Ohrenheilkunde, Kopf- und Halschirurgie, Universitätsklinikum Halle (Saale), Martin-Luther-Universität Halle-Wittenberg, Ernst-Grube-Str. 40, 06120 Halle (Saale), Deutschland; 2 Universitätsklinik und Poliklinik für Strahlentherapie, Universitätsklinikum Halle (Saale), Martin-Luther-Universität Halle-Wittenberg, Halle (Saale), Deutschland; 3 Universitätsklinik und Poliklinik für Neurochirurgie, Universitätsklinikum Halle (Saale), Martin-Luther-Universität Halle-Wittenberg, Halle (Saale), Deutschland

**Keywords:** Vestibularisschwannom, Reintonaudiogramm, Sprachverständlichkeit, Hörklassen, Akustikusneurinom, Vestibular schwannoma, Pure tone audiometry, Speech discrimination, Hearing classes, Acoustic neuroma

## Abstract

**Hintergrund:**

Die Klassifikation der Hörfunktion bei Patienten mit Vestibularisschwannom wird oft nach Gardner und Robertson (1988) oder Maßgaben der American Academy of Otolaryngology – Head and Neck Surgery (AAO-HNS, 1995) vorgenommen. Diesen Klassifikationssystemen liegen englische Sprachtestverfahren zugrunde. Eine deutschsprachige Entsprechung existiert nicht. Ziel der Arbeit ist die Untersuchung des Einflusses verschiedener Zielparameter auf die Hörklassifikation und die Ableitung einer Empfehlung für die Verwendung deutschsprachiger Testverfahren.

**Material und Methoden:**

Die auf englischsprachigen Testverfahren für die Sprachaudiometrie beruhenden Regeln wurden für deutsches Sprachmaterial fortgeschrieben. Darauf basierend wurde an einer Kohorte von 91 Patienten mit Vestibularisschwannom Reintonhörschwellen, Sprachverständlichkeitsschwelle und Sprachverständlichkeit bei verschiedenen Schalldruckpegeln gemessen und das Hörvermögen nach den Klassifizierungen Gardner und Robertson (1988) und AAO-HNS (1995) kategorisiert.

**Ergebnisse:**

Sowohl in der Gardner-Robertson-Klassifizierung als auch in der Klassifikation nach AAO-HNS ist die Anzahl der Patienten in den Hörklassen mit einer gut versorgbaren Hörschädigung (gemessen als Puretone-Average von drei (3PTA) oder vier Frequenzen (4PTA)) am höchsten, wenn der 3PTA_0,5;1;2_ _kHz_ verwendet wurde, gefolgt vom 4PTA_0,5;1;2;3_ _kHz_, 4PTA_0,5;1;2;4_ _kHz_ und 4PTA_0,5;1;2;“3”kHz_. Wird das maximale Sprachverstehen (Word Recognition Score, WRS_max_) anstelle des WRS bei 40 dB Sensation Level (WRS_40__SL_) verwendet, steigt die Anzahl der Patienten in den Hörklassen mit gut versorgbarer Hörschädigung unabhängig vom verwendeten Reintonhörschwellenmittelwert leicht.

**Schlussfolgerung:**

Die Klassifizierung der Hörfunktion nach Gardner und Robertson sowie AAO-HNS kann im deutschsprachigen Raum angewendet werden. Für die Bestimmung der Sprachverständlichkeit bzw. der maximalen Sprachverständlichkeit kann der Freiburger Einsilbertest verwendet werden.

## Abstract (en)

**Background:**

Hearing function in patients with vestibular schwannoma is often classified according to the Gardner and Robertson (1988) or the American Academy of Otolaryngology–Head and Neck Surgery (AAO-HNS, 1995) systems. These classification systems are based on English-language test procedures, there is no German-language equivalent. The aim of the study was to investigate the influence of various target parameters on hearing classification and to derive a recommendation for the use of German-language test methods.

**Materials and methods:**

The rules for speech audiometry based on English-language test procedures were applied to German speech test materials. In 91 patients with vestibular schwannoma, pure tone hearing thresholds, speech recognition thresholds, and speech discrimination at different sound pressure levels were measured. The patients were categorized according to the Gardner and Robertson and AAO-HNS classifications.

**Results:**

In both the Gardner–Robertson and the AAO-HNS classifications, the number of patients in the hearing classes with serviceable hearing function (measured as Pure Tone Average across three (3PTA) or four (4PTA) frequencies) was highest when using the 3PTA_0,5;1;2_ _kHz_ condition, followed by 4PTA_0,5;1;2;3_ _kHz_, 4PTA_0,5;1;2;4_ _kHz_, and 4PTA_0,5;1;2;“3”kHz_. If maximum word recognition score (WRS_max_) was used instead of word recognition 40 dB above the sensation level (WRS_40SL_), more patients were classified into the hearing classes with serviceable hearing function, irrespective of the mean pure tone hearing threshold.

**Conclusion:**

The Gardner–Robertson and AAO-HNS classifications can be used in German-speaking settings. The Freiburg monosyllabic test can be used to determine speech discrimination scores or maximum word recognition.

Cochleovestibuläre Schwannome sind gutartige Tumoren, die sich am häufigsten im inneren Gehörgang und im Kleinhirnbrückenwinkel (Vestibularisschwannome) und seltener auch im Innenohr (Cochlearisschwannome) entwickeln. Patienten mit Vestibularisschwannom entwickeln oft frühzeitig auditorische Symptome wie Hörverlust und Tinnitus. Vestibuläre Funktionsstörungen können mit zunehmender Tumorgröße auftreten.

Zur Beurteilung des Hörvermögens werden die Patienten häufig bezüglich ihrer Reintonhörschwellen und der Sprachverständlichkeit klassifiziert.

Die American Academy of Otolaryngology – Head and Neck Surgery (AAO-HNO) schlägt 4 Klassen (A–D) vor [1], wohingegen die Klassifikation nach Gardner und Robertson [10] 5 Klassen [1–5] unterscheidet und auf die Silverstein-Klassifikation (Klassen I–V) zurückgeht [35]. Im deutschsprachigen Raum wurde die „New-Hannover-Klassifikation“ mit 5 Stufen (H1–H5) entwickelt [29]. Die Tab. 1 und Abb. 1 zeigen die 4 Klassifikationssysteme im Vergleich zu den ebenfalls gelegentlich verwendeten Klassifikationen nach WHO [14] und der Global Burden of Disease (GBD) Hearing Loss Expert Group [37].

**Table 1:** 

**Silverstein (1986)** [35]			**Gardner-Robertson (1988)** [10]			**AAO-HNS (1995)** [1]			
**Klasse**	**3PTA**_**0,5;1;2**_ _**kHz**_ **(dB HL)**	**Sprachdiskrimination WRS**_**40SL**_** (%)**	**Klasse**	**3PTA**_**0,5;1;2**_ _**kHz**_ **(dB HL)**	**Sprachdiskrimination WRS**_**40SL**_** (%)**	**Klasse**	**4PTA**_**0,5;1;2;3**_ _**kHz**_ **(dB HL)**		**Sprachdiskrimination WRS**_**40SL**_** (%)**
I	0–30	70–100	1	0–30	100–70	A	≤30	*Und*	≥70
II	35–50	50–65	2	31–50	69–50	B	>30 und ≤50	*Und*	≥50
III	55–75	25–45	3	51–90	49–5	C	>50	*Und*	≥50
IV	80–100	0–20	4	>90	4–1	D	Alle Schwellen		<50
V	Nicht messbar	0	5	Keine Antwort	0				
**New Hannover (1997)** [29]			**WHO (1991)** [14]			**GBD Expert Group (2013)** [37]			
**Klasse**	**4PTA**_**0,5;1;2;3**_ _**kHz**_ **(dB HL)**	**Sprachdiskrimination WRS**_**max**_** (%)**	**Klasse**	**4PTA**_**0,5;1;2;4**_ _**kHz**_ **(dB HL)**		**Klasse**	**4PTA**_**0,5;1;2;4**_ _**kHz**_ **(dB HL)**		
H1	0–20	100–95	0	≤25	–	Normal	<20	–	
H2	21–40	95–70	1	26–40	–	Mild	20–34	–	
H3	41–60	65–40	2	41–60	–	Moderate	35–49	–	
H4	61–80	35–10	3	61–80	–	Mod. Sev.	50–64	–	
H5	>80	5–0	4	>80	–	Severe	65–79	–	
						Profound	80–94	–

**Figure 1:**
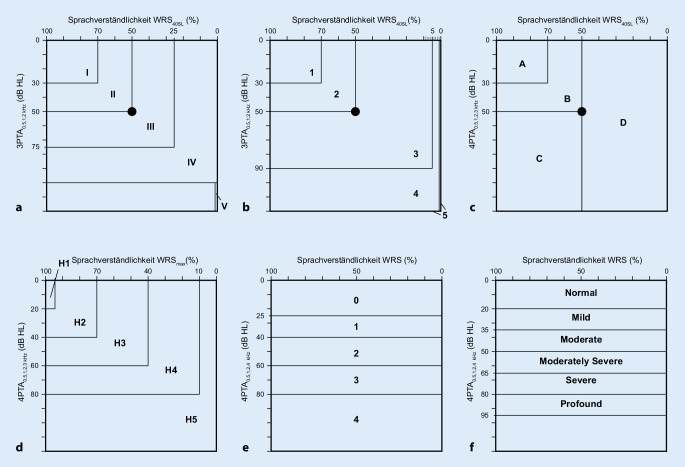

Die Einteilung der Hörfunktion in Klassen („Hörklassen“) erfolgt zunächst auf der Basis des Mittelwerts von Reintonhörschwellen („pure-tone average“, PTA), wobei die Definition des zugrunde legenden Mittelwerts verschieden ist. Die der Silverstein-Klassifikation zugrunde liegende Studie berechnet den Mittelwert der bei Frequenzen von 0,5; 1; und 2 kHz gemessenen Hörschwellen (3PTA_0,5;1;2_ _kHz_), definiert diesen Wert in der Klassifikation jedoch nicht eindeutig. Da sich die Gardner-Robertson-Klassifikation auf die Silverstein-Klassifikation bezieht und den PTA ebenfalls nicht eindeutig definiert, ist hier ebenfalls vom Mittelwert bei 0,5; 1 und 2 kHz (3PTA_0,5;1;2_ _kHz_) auszugehen. Die Gardner-Robertson-Klassifikation lässt die Sprachverständlichkeitsschwelle (SRT) alternativ zum PTA als Kriterium zu. Die AAO-HNO-Klassifizierung verwendet, wie die „New-Hannover-Klassifikation“, den PTA der Frequenzen 0,5; 1; 2 und 3 kHz (4PTA_0,5,1,3_ _kHz_). Eine Interpolation der Hörschwelle bei 3 kHz durch die Hörschwellen bei 2 und 4 kHz [1] ist zulässig, wenn diese nicht gemessen wurde.

Im zweiten Schritt wird die Sprachverständlichkeit als Entscheidungskriterium für die Hörklasse verwendet. Diese Messung beruht bei allen 3 Klassifikationssystemen auf der im angloamerikanischen Sprachraum etablierten Methode, die Sprachverständlichkeit bei einem Pegel von 40 Dezibel (dB) über der Sprachverständlichkeitsschwelle zu bestimmen (40-dB-Sensation-Level, SL, WRS_40SL_). In der AAO-HNS-Klassifikation wird dies noch genauer definiert und die beste Wortverständlichkeit bei Pegeln bis 40 dB SL oder bei maximal tolerierbarer Lautstärke (je nachdem, welcher Pegel kleiner ist) gemessen.

Aus beidem, der Sprachverständlichkeit und dem jeweiligen PTA, wird die Hörfunktion einer Hörklasse zugeordnet. Daraus ergeben sich die in Abb. 1 dargestellten Nomogramme. Gelegentlich wird auch die nur auf dem 4PTA_0,5;1;2;4_ _kHz_ basierende Klassifikation nach WHO-Kriterien, welche sich in ihrem eigentlichen Anwendungszweck allerdings auf das bessere Ohr bezieht, verwendet [14, 28, 40]. Diese sowie die Klassifikation der GBD Expert Group beschreiben das Hörvermögen allein anhand der Reintonhörschwelle und ordnen lediglich passende Kategorien der Sprachverständlichkeit zu [14, 37]. Bei der Betrachtung des Hörerhalts nach Cochleaimplantation wird eine nur auf den Reintonhörschwellen basierende Klassifikation verwendet [36], welche durch das Fehlen der sprachaudiometrischen Daten jedoch nicht für Patienten mit Vestibularisschwannom verwendet werden kann.

Ziel der Klassifikationssysteme nach Silverstein, Gardner-Robertson und AAO-HNS ist auch die Stratifizierung der Patienten anhand der Grenzen 50 % Sprachverständlichkeit und 50 dB Hörverlust (HL) im PTA. Diese auf Wade und House [39] zurückgehende Regel (50/50-Regel) trennt die Klassen mit für den Alltag funktionalem Hörvermögen von den Klassen ohne versorgbare („serviceable“) Hörfunktion. Diese Unterscheidung wurde für die Stratifizierung von Studienteilnehmern [2, 8, 18, 22, 26], die Entscheidung für eine Therapie oder den operativen Zugang verwendet [30, 31, 41]. Meyer et al. [19] schlagen sogar vor, allein nach der Sprachverständlichkeit zu klassifizieren.

Die Klassifikationssysteme sind in ihrer Anwendung nicht immer einheitlich. So soll in der Silverstein-Klassifikation das Hören in die schlechtere („poorer“) Klasse fallen, wenn die Zuordnung nach PTA und Sprachverständlichkeit unterschiedliche Klassen ergibt. Die Gardner-Robertson-Klassifikation fordert in diesem Fall die Einordnung in die niedrigere („lower“, d. h. bessere) Klasse, was einen Widerspruch ergibt. Die AAO-HNS-Klassifikation enthält keine Vorgabe für diesen Fall. Die Gardner-Robertson-Klassifizierung unterscheidet zudem beim PTA zwischen maximalem Hörverlust („max. loss“) und nicht messbaren Hörschwellen („no response“), was eine eindeutige Klassifizierung in manchen Fällen nicht ermöglicht. Bei der Gardner-Robertson-Klassifizierung wird alternativ zum PTA die Messung des SRT zugelassen. Beide Messgrößen korrelieren nur bedingt und beruhen auf in unterschiedlichen Einheiten gemessenen Stimulationspegeln (dB HL vs. dB SPL). Somit ergeben sich möglicherweise mehrdeutige Einordnungen.

Im deutschsprachigen Raum werden Testverfahren für die Sprachaudiometrie bei der Diagnostik von Vestibularisschwannomen verwendet, die sich neben dem Sprachmaterial in ihrer Diskriminationsfunktion und Testparametern von den englischsprachigen, die den o. g. Klassifizierungen zugrunde liegen, unterscheiden. So wird im Freiburger Einsilbertest pro Stimulationspegel in der Regel eine Liste mit 20 Wörtern verwendet, wohingegen die Klassifizierungen aus dem angloamerikanischen Raum auf der genaueren Messung mit 50 Wörtern je Liste (z. B. PB-50-Test, [9]) beruhen [27]. Die im deutschsprachigen entwickelte „New Hannover-Klassifikation“ misst der Sprachverständlichkeit eine untergeordnete Rolle zu. So wird das zu verwendende Testmaterial nicht angegeben [37], wenngleich aus den Abbildungsformatierungen die Verwendung des Freiburger Einsilbertests geschlossen werden kann. Hier wird, im Gegensatz zur Verwendung des WRS_40SL_, die maximale Sprachverständlichkeit (WRS_max_) gemessen, aber, im Vergleich zur Klassifizierung nach der Hörschwelle gemäß Gardner-Robertson-Verfahren, immer die der geringeren Hörfunktion entsprechende Klasse gewählt.

Zur Klassifikation des Hörvermögens wird im deutschsprachigen Raum häufig die Hörschwelle bei 4 kHz in die Berechnung des PTA einbezogen, so z. B. bei der Hörsturzdiagnostik [23] der Ohrchirurgie [43] und konsequenterweise auch bei der Diagnostik von Vestibularisschwannomen [29, 34]. Da nach den Klassifikationen nach Silverstein, Gardner-Robertson, AAO-HNS, aber auch nach der „New Hannover-Klassifikation“ die Hörschwelle bei 4 kHz nicht betrachtet wird, ist unklar, wie sich deren Einschluss oder die von der AAO-HNS vorgeschlagene Interpolation der Hörschwelle bei 3 kHz [1] auf die Klassifikation auswirkt.

Um die Vergleichbarkeit zu englischsprachigen Arbeiten zu ermöglichen und das für den Alltag der Patienten relevante Sprachverstehen einzuschließen, werden auch im deutschsprachigen Raum Patienten mit Vestibularisschwannom häufig unter Anwendung der beschriebenen Klassifikationen analysiert [4, 16, 17, 24, 25, 29, 32, 33]. Die zugrunde liegenden Parameter, d. h., die Wahl der PTA-Frequenzen und des Sprachmaterials, sind dabei sehr heterogen oder gar nicht angegeben. Eine Adaptationsregel der bestehenden englischsprachigen Klassifikationssysteme für die Verwendung deutschen Testmaterials fehlt.

In dieser Arbeit soll das Hörvermögen eines exemplarischen, eigenen Patientenkollektivs mit Vestibularisschwannom entsprechend Gardner-Robertson (1988) und AAO-HNS (1995) klassifiziert werden und dabei deutschsprachiges Testmaterial eingesetzt und zusätzlich verschiedene Frequenzbereiche für den Reintonhörschwellenmittelwert verwendet werden. Die Auswirkungen auf die so adaptierten Klassifizierungen werden verglichen und diskutiert. Daraus soll eine Empfehlung zur Klassifizierung von Hörstörungen bei Patienten mit Vestibularisschwannom nach Gardner-Robertson (1988) und AAO-HNS (1995) bei Verwendung deutschsprachiger Testverfahren abgeleitet werden.

## Methodik

Die Hörfunktion einer exemplarischen Kohorte von Patienten mit Vestibularisschwannom aus der klinischen Routine soll nach verschiedenen Berechnungsmethoden in Hörklassen kategorisiert werden. Für die Fallzahlschätzung wurde die Sprachverständlichkeit als Endpunkt mit der im Vergleich zum PTA größeren Streuung betrachtet. Die zu erwartende Effektstärke wurde unter Annahme einer der Klassenbreite entsprechenden Standardabweichung (30 %) und eines relevanten Mittelwertunterschieds von 10 % nach Cohen mit r = 0,16 geschätzt (Cohen-d = 0,3). Unter Annahme eines Alphaniveaus von 0,05 und einer Power von 0,6 ergibt sich daraus eine Stichprobengröße von 90. Somit wurden die Daten der im Zeitraum Januar 2016 bis März 2020 zur Diagnostik eines Vestibularisschwannoms audiologisch und neurootologisch diagnostizierten 91 Patienten für diese Studie ausgewertet.

Vor der Therapie wurden die Reintonhörschwellen für Luft- und Knochenleitung bei den Frequenzen 0,25; 0,5; 1; 2; 3; 4 und 8 kHz gemessen. Patienten mit Schallleitungsschwerhörigkeit waren nicht in dieser Gruppe.

Der Freiburger Zahlentest wurde verwendet, um die 50%-Sprachverständlichkeitsschwelle (SRT) zu messen. Diese wurde in einigen Fällen durch Interpolation und Rundung auf 5 dB SPL bestimmt. Mit dem Freiburger Einsilbertest wurde die Sprachverständlichkeit in Ruhe bei einem Schalldruckpegel (SPL) von 50, 65, 80, 95 und 110 dB bestimmt. Die Sprachverständlichkeit bei 40 dB über dem SRT (WRS_40SL_) und die maximale Sprachverständlichkeit (WRS_max_) wurden ebenfalls mit dem Freiburger Einsilbertest wie in der klinischen Routine üblich bestimmt. Alle audiometrischen Messungen wurden in einer schallgedämmten Kabine nach DIN ISO 8253 mit Kopfhörern durchgeführt. Die Gegenseite wurde, wenn nötig, mit weißem Rauschen maskiert.

Aus den Hörschwellen für Luftleitung wurden die Mittelwerte 3PTA_0,5;1;2_ _kHz_, 4PTA_0,5;1;2;3_ _kHz_, 4PTA_0,5;1;2;4_ _kHz_ und durch Ersetzen der Hörschwelle bei 3 kHz durch den Mittelwert der Schwellen bei 2 kHz und 4 kHz der 4PTA_0,5;1;2;“3”kHz_ gebildet.

Basierend auf den so gemessenen jeweiligen PTA und WRS wurden die Patienten entsprechend der Regeln für die Klassifizierungen nach Gardner und Robertson (1988) und AAO-HNS (1995) kategorisiert.

## Ergebnisse

Eingeschlossen werden konnten 91 Patienten (28 weiblich, 65 männlich) mit unilateralem Vestibularisschwannom im Alter von 18–77 Jahren (Mittelwert; MW: 51,6 Jahre). Die Tumorausdehnung war über alle Klassen nach Koos verteilt (I: 13, II: 28, III: 29, IV: 21). Davon hatten 47 Patienten den Tumor auf der rechten und 44 auf der linken Seite. Die Reintonhörschwelle des Gegenohrs (4PTA_0,5;1;2;4_ _kHz_) war über alle Koos-Klassen etwa gleich (12,6–18,9 dB HL) und im Mittel 17,5 dB HL (SD: 11 dB HL).

Die Tab. 2 zeigt die Hörklasseneinteilung nach Gardner-Robertson und AAO-HNS für das untersuchte exemplarische Patientenkollektiv mit Vestibularisschwannom. Sowohl nach der Gardner-Robertson-Klassifizierung als auch nach der Klassifikation nach AAO-HNS ist die Anzahl der Patienten in den Hörklassen mit gut versorgbarer Hörfunktion (1, 2 bzw. A, B) abhängig von der jeweiligen zugrunde liegenden Berechnungsmethode, aber für jede Berechnungsmethode bei beiden Klassifizierungen gleich. Hält man die Wahl des WRS konstant, sind die meisten Patienten in den Hörklassen mit nutzbarer Hörfunktion, wenn der 3PTA_0,5;1;2_ _kHz_ verwendet wurde, gefolgt vom 4PTA_0,5;1;2;3_ _kHz_, 4PTA_0,5;1;2;4_ _kHz_ und 4PTA_0,5;1;2;“3”kHz_. Wird der WRS_max_ anstelle des WRS_40SL_ verwendet, steigt die Anzahl der Patienten in den Hörklassen mit nutzbarer Hörfunktion unabhängig vom verwendeten Reintonhörschwellenmittelwert leicht.

**Table 2:** 

	WRS_40SL_				WRS_max_			
Klasse	3PTA_0,5;1;2_ _kHz_	4PTA_0,5;1;2;3 kHz_	4PTA_0,5;1;2;“3”_ _kHz_	4PTA_0,5;1;2;4 kHz_	3PTA_0,5;1;2 kHz_	4PTA_0,5;1;2;3 kHz_	4PTA_0,5;1;2;“3”_ _kHz_	4PTA_0,5;1;2;4 kHz_
*Gardner-Robertson (1988)*								
1	**31**	27	27	26	31	27	27	26
2	**27**	30	27	29	29	31	28	30
3	**20**	20	24	23	19	20	24	23
4	**0**	1	0	0	0	1	0	0
5	**13**	13	13	13	12	12	12	12
1–2	**58**	57	54	55	60	58	55	56
3–5	**33**	34	37	36	31	33	36	35
*AAO-HNS (1995)*								
A	31	**27**	27	26	31	27	27	26
B	27	**30**	27	29	29	31	28	30
C	7	**8**	11	10	8	10	13	12
D	26	**26**	26	26	23	23	23	23
A–B	58	**57**	54	55	60	58	55	56
C–D	33	**34**	37	36	31	33	36	35

**Figure 2:**
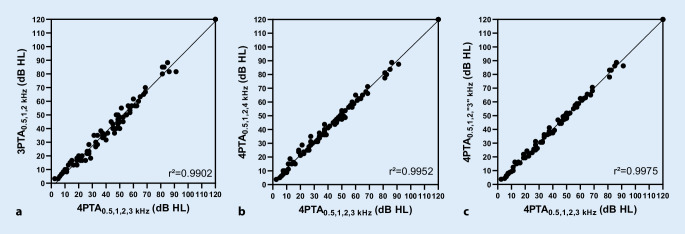

Die Abb. 2 zeigt die Korrelation des 4PTA_0,5;1;2;3_ _kHz_ mit dem 3PTA_0,5;1;2_ _kHz_, 4PTA_0,5;1;2;3,4_ _kHz_ und 4PTA_0,5;1;2;“3”kHz_. Der 3PTA_0,5;1;2_ _kHz_ hat einen „offset“ von −3,18 dB im Vergleich zum 4PTA_0,5;1;2;3_ _kHz_ bei einer Steigung von 1,016 dB/dB. Bei Einschluss der 4 kHz (4PTA_0,5;1;2;4_ _kHz_) besteht ein „offset“ von 1,88 dB HL (Steigung 0,978 dB/dB), bei Interpolation des 3 kHz-Werts (4PTA_0,5;1;2;“3”kHz_) von 0,354 dB HL (Steigung 0,995 dB/dB) im Vergleich zum 4PTA_0,5;1;2;3_ _kHz_. Die Zusammenhänge sind hochsignifikant linear (*p* < 0,001, *r*^2^ = 0,99).

**Figure 3:**
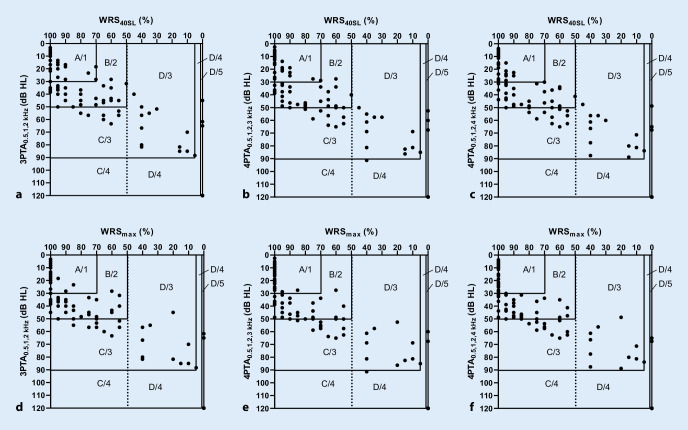

Die Abb. 3 zeigt die Zuordnung der Patienten zu den Hörklassen nach Gardner-Robertson und AAO-HNS bei Verwendung der jeweiligen PTA und WRS als Kriterien.

## Diskussion

### Hörklassifikation bei Verwendung deutschsprachiger Testverfahren

#### Einfluss der Reintonschwellenaudiometrie

Die Ergebnisse an der exemplarisch untersuchten Kohorte zeigen, dass die Zuordnung in eine Hörklasse nach Gardner-Robertson oder AAO-HNS in allen Fällen durchgeführt werden konnte. Die Hörklasse hängt jedoch vom jeweiligen verwendeten PTA ab. Eine Veränderung der Klassifizierung hin zu Klassen mit weniger Hörfunktion ergab sich im Vergleich zum 3PTA_0,5;1;2_ _kHz_, wenn der 4PTA_0,5;1;2;3_ _kHz_ oder 4PTA_0,5;1;2;4_ _kHz_ verwendet wurde, jedoch insbesondere mit dem 4PTA_0,5;1;2;“3”kHz_, bei dem die Hörschwelle bei 3 kHz durch die Werte bei 2 kHz und 4 kHz interpoliert wurde, wie von Gurgel et al. [11, 12] vorgeschlagen und auch in Metaanalysen praktiziert [15]. Die Messung des 3PTA_0,5;1;2_ _kHz_ und auch des 4PTA_0,5;1;2;3_ _kHz_ ist im deutschsprachigen Raum eher unüblich, wird jedoch auch außerhalb des angloamerikanischen Sprachraums gelegentlich für die Einteilung in Hörklassen verwendet [42]. Da die Klassifikationen nach Gardner-Robertson und AAO-HNO jedoch auf dem PTA über die Frequenzen 0,5; 1 und 2 kHz bzw. 0,5; 1; 2 und 3 kHz basieren, sollten dennoch diese im deutschsprachigen Raum weniger verwendeten Mittelwerte für die entsprechende Klassifizierung verwendet werden. Dies schließt explizit die Notwendigkeit der Hörschwellenmessung bei 3 kHz ein.

Unklar bleibt in allen Klassifikationssystemen die Frage, wie mit Patienten umgegangen werden soll, die zusätzlich zu der auf ihrer Tumorerkrankung beruhenden Schallempfindungsschwerhörigkeit eine Schallleitungsschwerhörigkeit haben. Hier wäre zu empfehlen, die Knochenleitungshörschwelle für die Klassifizierung zu verwenden.

#### Einfluss des Sprachmaterials und des Sprachverstehensmaßes

Die Klassifizierungen nach Gardner-Robertson und AAO-HNS basieren auf der Messung der Sprachverständlichkeit mit nicht näher spezifizierten englischsprachigen Testverfahren. Hier stellt sich nun die Frage nach der Übertragbarkeit auf deutschsprachige Patienten und Testverfahren. Studien zu Patienten mit Vestibularisschwannom verwenden im deutschsprachigen Raum den Freiburger Einsilbertest [4, 17, 25, 32] oder spezifizieren das für die Klassifikation verwendete Testmaterial nicht näher [16, 24, 29, 33]. Eine Angabe über den verwendeten Stimulationspegel fehlt meist.

Eine informelle Umfrage unter 6 Zentren für die Therapie von Vestibularisschwannomen in Deutschland ergab ein ähnliches Bild: Es wird der Freiburger Einsilbertest verwendet und die maximale Sprachverständlichkeit, d. h., ohne eine bestimmte Stimulationspegelvorgabe, bestimmt. Für die Bestimmung der Sprachverständlichkeitsschwelle wird der Freiburger Zahlentest verwendet.

Nach den in dieser Arbeit erhaltenen Ergebnissen ergeben beide Klassifizierungen (nach Gardner-Robertson und AAO-HNS) eine geringfügig höhere (bessere) Klasse, wenn der WRS_max_ anstelle des WRS_40SL_ verwendet wird und bestätigt damit frühere Arbeiten mit deutschsprachigem Testmaterial [29]. Die Klassifizierungsmethoden nach Gardner-Robertson und AAO-HNS schreiben kein spezifisches Sprachtestmaterial vor. Daher wird die Übertragung in den deutschsprachigen Raum unproblematisch gesehen, wenn, wie bei der Indikation für die Cochleaimplantatversorgung, Testmaterial mit ähnlichen Diskriminationsfunktionen verwendet wird [13]. Das zugrunde liegende englischsprachige Sprachmaterial ist nicht vorgegeben. Wird der häufig verwendete PB-50-Test [9] für den Vergleich zugrunde gelegt, wäre dies beim Freiburger Einsilbertest der Fall.

Die im deutschsprachigen Raum aus dem Sprachaudiogramm bei Lärmschwerhörigkeit nach Boenninghaus und Röser [6] sowie nach der Versorgungsmedizin-Verordnung [7] ermittelten prozentualen Hörverluste sind u. a. wegen des bei Vestibularisschwannomen häufig zu beobachtenden „Roll-over-Effekts“ im Sprachaudiogramm oder die integrierte Betrachtung beider Ohren für die Fragestellung dieser Arbeit nicht geeignet.

Die Ergebnisse zeigen eine große Streuung der Sprachverständlichkeit in Abhängigkeit von PTA, insbesondere bei moderaten PTA. Weil die Sprachverständlichkeit jedoch das für den Alltag der Patienten relevantere Maß ist, sollten Beurteilungen der Hörfunktion deren Messung stets miteinbeziehen.

Um eine Vergleichbarkeit der Daten innerhalb des deutschsprachigen Raums zu erzielen, sind einheitliche Methoden notwendig. Zunächst ergeben sich für die Einteilung in Hörklassen nach Gardner-Robertson und AAO-HNS die in Tab. 3 gezeigten Empfehlungen für die Anwendung dieser Klassifizierungsmethoden im deutschsprachigen Raum. Der WRS_max_ sollte anstelle des WRS_40SL_ verwendet werden. Wird berücksichtigt, dass die Empfehlung der AAO-HNS die aktuellere Methode des angloamerikanischen Sprachraums ist, sollte diese – entsprechend für den deutschsprachigen Raum – idealerweise ebenfalls angewendet werden.

**Table 3:** 

Zielgröße	Messverfahren	Parameter
Mittlere Reintonschwelle (PTA)	Reintonaudiogramm für Luftleitung	Gardner-Robertson: 0,5; 1; 2 kHz; AAO-HNS: 0,5; 1; 2; 3 kHz
Sprachverständlichkeitsschwelle (SRT)	Freiburger Zahlentest	50%-Schwelle
Maximale Sprachverständlichkeit (WRS_max_)	Freiburger Einsilbertest	Mehrere Pegel, maximale Einsilberverständlichkeit

### Notwendigkeit für weitere Zielparameter

Die interdisziplinäre Therapie von Vestibularisschwannomen hat in der Vergangenheit einen Paradigmenwechsel hin zu strukturerhaltender Therapie mit dem Ziel des Erhalts und der Wiederherstellung der Hörfunktion erfahren. Um die Veränderung des Hörvermögens genauer beurteilen zu können, sind kontinuierliche Messgrößen, wie sie z. B. in Hörsturzstudien [23] und zunehmend auch der Radiatio [21] verwendet werden, geeigneter als die Klassifizierung in nur wenige Klassen. Die GBD Expert Group geht ebenfalls den Weg hin zu einer feingliedrigeren Klassifizierung [37].

In Studien mit audiologischen Fragestellungen ist die Messung der ton- und sprachaudiometrischen Endpunkte als kontinuierliche Variable etabliert und liefert weitaus mehr Informationen als eine Angabe der Hörklasse. Diese kann dann im Sinne einer Datenreduktion ggf. später durchgeführt werden und ermöglicht – wenn z. B. bei Patienten mit Vestibularisschwannom nach Gardner-Robertson oder AAO-HNS vorgenommen – den internationalen Vergleich. Die Verwendung kontinuierlicher Variablen verringert zudem die Anzahl in Studien zur Hördiagnostik und -therapie einzuschließender Patienten deutlich im Vergleich zu groben Klassifizierungen [23], wie sie z. B. von Gurgel et al. vorgeschlagen wurden [11].

Die Möglichkeit einer Cochleaimplantation nach Therapie eines Vestibularisschwannoms [3–5, 15, 17, 20, 34] erfordert ohnehin eine umfangreiche Diagnostik des Hörvermögens nach audiologischen Standards. Dazu ist es notwendig, das Hörvermögen sowohl auf Basis der Reintonaudiometrie als auch auf Basis der Sprachaudiometrie zu bestimmen. Eine alleinige Bestimmung der Reintonhörschwelle [38, 40] würde für die Indikation eines Cochleaimplantats oder auch eines Hörgeräts nicht ausreichen. Daher wird für die weitere audiologische Diagnostik die Messung eines vollständigen Ton- und Sprachaudiogramms empfohlen. Wenn die Messung der Hörschwelle bei 3 kHz eingeschlossen ist, kann zusätzlich zur detaillierten audiologischen Diagnostik auch durch Anwendung der bestehenden Klassifikationen die internationale Vergleichbarkeit gewährleistet werden.

## Fazit für die Praxis

- Die Klassifizierungen nach Gardner-Robertson und AAO-HNS können im deutschsprachigen Raum für die Untersuchung der Hörfunktion bei Patienten mit Vestibularisschwannom angewendet werden.
- Für die Bestimmung der Reintonhörschwelle kann der Hörschwellenmittelwert über die Frequenzen 0,5; 1 und 2 kHz bzw. 0,5; 1; 2 und 3 kHz verwendet werden.
- Für die Bestimmung der maximalen Sprachverständlichkeit sollte der Freiburger Einsilbertest verwendet werden.
- Die maximale Sprachverständlichkeit (WRS_max_) sollte bestimmt und der Klassifikation zugrunde gelegt werden.
